# Supplementary figures and images for: Characterization of eclosion hormone receptor function reveals differential hormonal control of ecdysis during Drosophila development
Source: PLoS Genet. 2025 Aug 20;21(8):e1011672. doi: 10.1371/journal.pgen.1011672 (PMC12393706; doi:10.1371/journal.pgen.1011672)

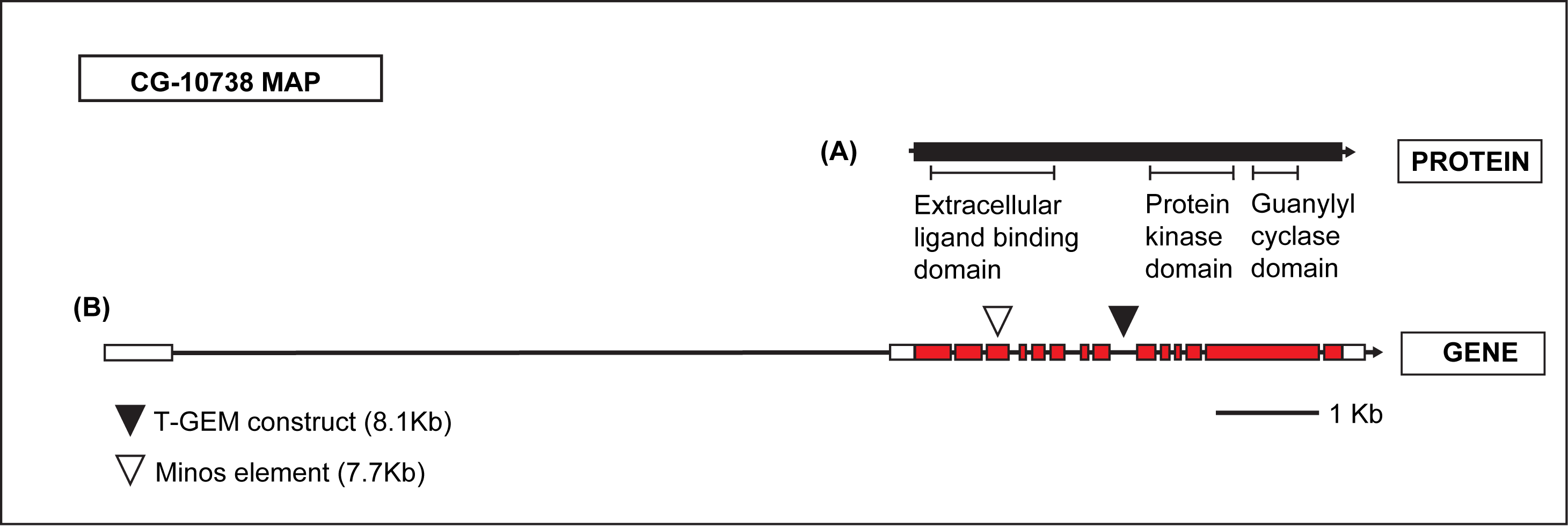

Supplement: S1 Fig — Schematic representation of the CG10738 protein (A) and gene (B). (A) Protein symbolized as a black box includes three domains predicted by InterPro. (B) Gene map shows the location of insertions used here, which include the EHR-GAL4 (T-GEM construct) and a Minos insert. Non-coding regions are indicated as white boxes, coding regions as red boxes, and introns as black lines. Scale bar: 1 Kb. (TIF) [file pgen.1011672.s001.tif]

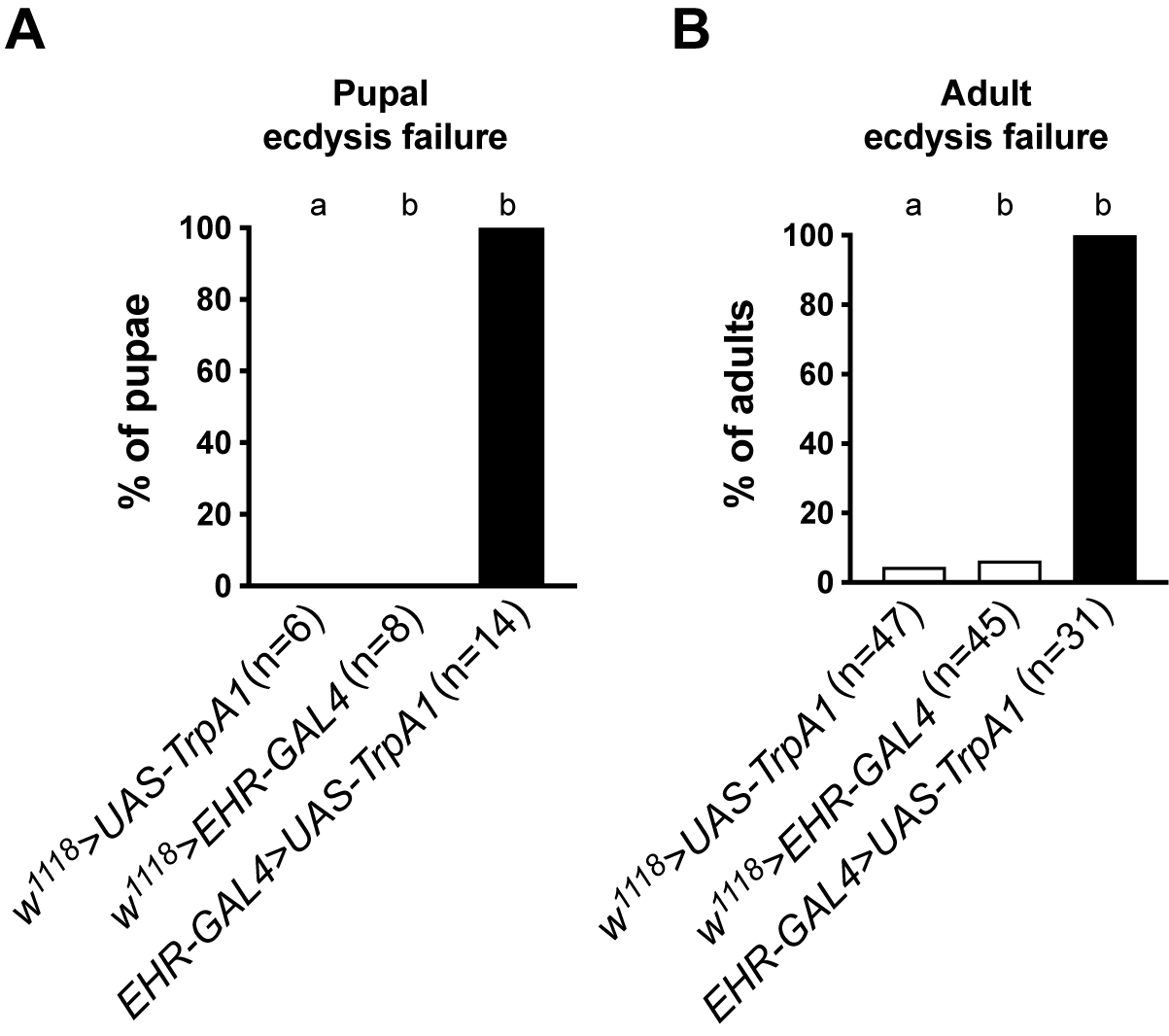

Supplement: S2 Fig — Percentage of animals that failed to complete pupal ecdysis (A), and adult eclosion (B), after activation of EHR-expressing cells using UAS-TrpA1 for 2h before ecdysis. Statistical analyses were performed using Fisher’s exact test, and significant differences are indicated by letters (see results in S3 Table). (TIF) [file pgen.1011672.s002.tif]

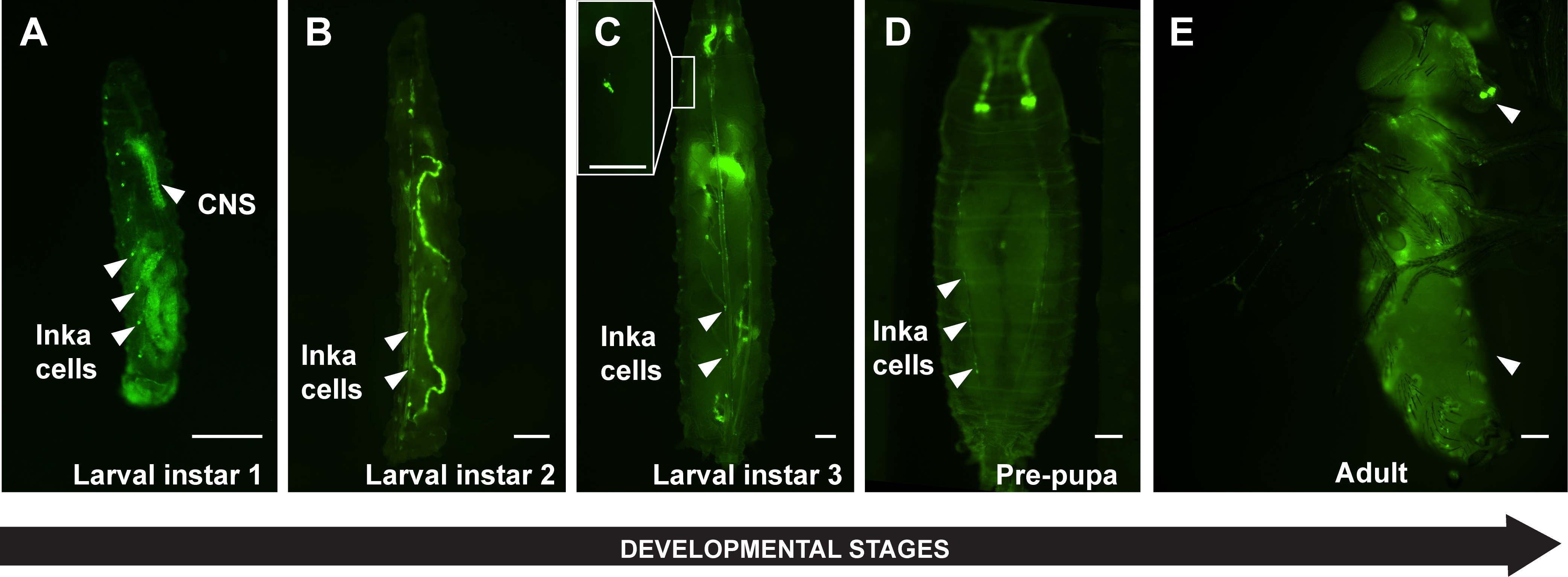

Supplement: S3 Fig — Whole body pattern of EHR expression visualized with GFP (EHR > GFP) at the first (A), second (B), and third (C) larval instar; at the P3 pre-pupal stage (D); and at the pharate adult stage (E). Insert in (C) shows EHR expression in some cells of the body wall; white arrowheads in (E) point the EHR expression in the proboscis (top) and in dorsal bands of cells of the body wall (bottom). Scale bar 200 μm for panels and (A-E), and 100 μm for insert in (C). (TIF) [file pgen.1011672.s003.tif]

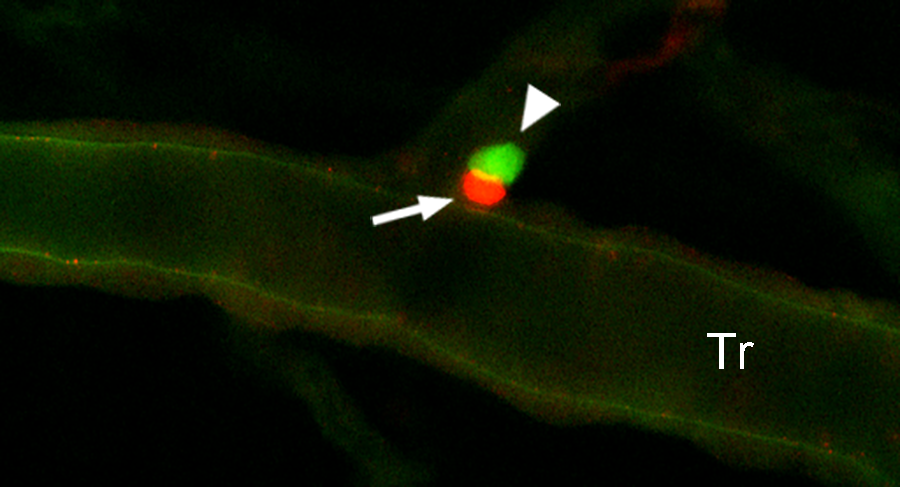

Supplement: S4 Fig — ETH receptor (ETHR) expression (white arrowhead) in third larval instar tracheal preparation visualized using a membrane-bound GFP (ETHR>mCD8-GFP, in green), together with ETH-immunoreactivity (in red)(white arrow). ETHR-expressing cell is immediately adjacent to the Inka cell. Tr: dorsal tracheal trunk. (TIF) [file pgen.1011672.s004.tif]
