# Supplementary material for: Characterization of eclosion hormone receptor function reveals differential hormonal control of ecdysis during Drosophila development
Source: PLoS Genet. 2025 Aug 20;21(8):e1011672. doi: 10.1371/journal.pgen.1011672 (PMC12393706; doi:10.1371/journal.pgen.1011672)
Supplement: S1 Table — (DOCX) [file pgen.1011672.s006.docx]

**S1 Table: List of all the strains used and their source.**

| **GENOTYPE** | **DESCRIPTION** | **SOURCE^1^** |
| --- | --- | --- |
| *w^1118^* (control) | *white* mutant; genetic background of most transgenic animals used here. | Lab stock |
| UAS-EHR (CG10738) | EH Receptor | This study |
| EHR-GAL4 (CG10738) | GAL4 driver for EH Receptor | This study |
| *Tubulin-*GAL4 | GAL4 driver for the microtubule tubulin | BL5138 |
| *n-syb-*GAL4 | GAL4 driver for all neurons neuronal synaptobrevin | BL51942 |
| *eth-*GAL4 | GAL4 driver for ETH (Inka cells) | Ewer lab |
| *386Y-*GAL4 | GAL4 driver for peptidergic and secretory cells | Supplied by P. Taghert |
| *burs*-GAL4 | GAL4 driver for bursicon peptide | Lab stock |
| *EH^ups^-*GAL4 | GAL4 driver for EH peptide (Vm neurons) | Supplied by S. McNabb |
| *EH^pan^-*GAL4 | GAL4 driver for Vm and new EH neurons | Supplied by B. White |
| *Mimic{ETHR}-*GAL4 | GAL4 driver for ETH Receptor | BL32718 |
| *VGlut-*GAL4 | GAL4 driver for Vesicular Glutamate transporter 1 | BL60312 |
| *tsh-*GAL4 | GAL4 driver for t-shirt (trunk patterning expression) | Supplied by C. Wegener |
| *btl*-GAL4 | GAL4 driver for breathless (tracheal cells) | BL8807 |
| *ChAT*-GAL4 | GAL4 driver for cholinergic neurons | BL60317 |
| *c929*-GAL4 | GAL4 driver for peptidergic neurons | Lab stock |
| *repo*-GAL4 | GAL4 driver for glial cells | Supplied by R. Jackson |
| *c164*-GAL4 | GAL4 driver for motoneurons and other neurons | Supplied by V. Budnik |
| *DDC*-GAL4 | GAL4 driver for dopaminergic and serotonergic neurons | BL7009 |
| *CCAP*-GAL4 | GAL4 driver for CCAP peptide | Ewer lab |
| *trhn*-GAL4 | GAL4 driver for serotoninergic cells | BL84694 |
| *ple*-GAL4 | GAL4 driver for dopaminergic cells | BL86289 |
| *sNPF*-GAL4 | GAL4 driver for sNPF peptide | BL84706 |
| *ETHR*-GAL4 | GAL4 driver for ETH-Receptor | Supplied by B. White |
| *CCAP-R*-GAL4 | GAL4 driver for CCAP-Receptor | Supplied by B. White |
| *rk*-GAL4 | GAL4 driver for bursicon receptor (*ricket*) | Supplied by B. White |
| *GAD1*-GAL4 | GAL4 driver for glutamatergic cells | Supplied by P. Taghert |
| *nompC*-GAL4 | GAL4 driver for peripheral sensory neurons | Supplied by B. White |
| *Gr66a*-GAL4 | GAL4 driver for gustatory neurons | BL28801 |
| *109(2)80-*GAL4 | GAL4 driver for dendritic neurons, oenocytes and chordotonal organs | BL8769 |
| *Ir20a*-GAL4 | GAL4 driver for gustatory neurons and adult abdominal segments | BL60694 |
| *Ir8a*-GAL4 | GAL4 driver for ionotropic glutamate receptor | BL41731 |
| *Smid c161*-GAL4 | GAL4 driver for Bolwig's nerve, chordotonal organs and imaginal discs) | BL27893 |
| *Ir25a*-GAL4 | GAL4 driver for sensory neurons | BL41728 |
| *Ir7g*-GAL4 | GAL4 driver for gustatory receptor neurons | BL81223 |
| *Ir40a*-GAL4 | GAL4 driver for hydrosensory neurons | BL41727 |
| 410-GAL4 | GAL4 driver for class I dendritic arbor neurons. | BL63298 |
| EHR-GAL4DBD or -p65AD | Split-GAL4 driver for EHR | This study |
| EH^pan^-p65AD | Split-GAL4 driver for EH peptide (Vm and new targets) | Supplied by B. White |
| ETHR-p65AD | Split-GAL4 driver for ETH Receptor | Supplied by B. White |
| ETHRA-p65AD | Split-GAL4 driver for ETHR isoform A | Supplied by B. White |
| ETHRB-p65AD | Split-GAL4 driver for ETHR isoform B | Supplied by B. White |
| Tub-DBD or -AD | Split-GAL4 driver for microtubule tubulin | BL60298 |
| Tub-DBD or -AD | Split-GAL4 driver for microtubule tubulin | BL60295 |
| *elav*-AD | Split-GAL4 driver for all neurons | Supplied by B. White |
| CCAP-GAL4DBD | Split-GAL4 driver for CCAP peptide | Supplied by B. White |
| CCAP-R-p65AD | Split-GAL4 driver for CCAP receptor | Supplied by B. White |
| *burs-*DBD | Split-GAL4 driver for bursicon peptide | Supplied by B. White |
| *rk*-AD | Split-GAL4 driver for bursicon receptor | Supplied by B. White |
| VGlut-p65AD | Split-GAL4 driver for Vesicular Glutamate transporter 1 | Supplied by B. White |
| ChAT-p65AD | Split-GAL4 driver for choline acetyltransferase | Supplied by B. White |
| Tub-GAL80ts | GAL80 line temperature sensitive | Lab stock |
| UAS-EHR RNAi | RNAi against EHR | BL38346 |
| UAS-EHR RNAi | RNAi against EHR | BL57318 |
| UAS-EHR RNAi | RNAi against EHR | BL60439 |
| UAS-EHR RNAi | RNAi against EHR | BL28580 |
| UAS-EHR RNAi | RNAi against EHR | NIG10738-R1, III |
| UAS-EHR RNAi | RNAi against EHR | NIG10738-R2, II |
| UAS-ETHR RNAi | RNAi against ETHR | VDRC42717 |
| *Df(3L)exel9017* | Genetic deletion that includes CG10738 | BL7934 |
| Minos(EHR) | Minos insertion in CG10738 gene | BL24564 |
| Mimic(EHR) | Mimic insertion in CG10738 gene | BL35974 |
| UAS-GFP | Green Fluorescent Protein (GFP) | BL6874 |
| UAS-GFP nuclear | Nuclear GFP | BL4775 |
| UAS-RFP nuclear | Nuclear Red Fluorescent Protein (RFP) | Lab stock |
| UAS-mCherry | Monomeric cherry fluorescent protein | BL27391 |
| UAS-esg-GFP | escargot (esg)-GFP fusion protein | Supplied by B. White |
| UAS-*reaper* | Apoptotic factor | BL5824 |
| UAS-*Kir.2.1* | Inwardly rectifying potassium channel | Supplied by B. White |
| UAS-GCaMP6s | Calcium sensitive GFP | BL42749 |
| UAS-*TrpA1* | Transient receptor potential cation channel | BL26263 |
| UAS-d*cr2* | Dicer-2 | Lab stock |

^1^BL: Bloomington *Drosophila* stock center (Bloomington, USA); NIG: Fly stocks of National Institute of Genetics (Mishima, Japan); VDRC: Vienna Drosophila Resource Center (Vienna, Austria).
