# Supplementary material for: Characterization of eclosion hormone receptor function reveals differential hormonal control of ecdysis during Drosophila development
Source: PLoS Genet. 2025 Aug 20;21(8):e1011672. doi: 10.1371/journal.pgen.1011672 (PMC12393706; doi:10.1371/journal.pgen.1011672)
Supplement: S2 Table — Phenotype of RNAi lines for EHR expressed using tubulin-GAL4. (PDF) [file pgen.1011672.s007.pdf]

Table S2: EHR RNAi screening. Phenotype of RNAi lines for EHR expressed using *tubulin*-GAL4.

RNAi line	Phenotype
BL38346 (Bloomington, USA)	Normal pupal ecdysis. Some adults with wing expansion defects
BL57318 (Bloomington, USA)	Crosses produced few animals; no adult eclosed
BL60439 (Bloomington, USA)	Some pupae with ecdysial problems
BL28580 (Bloomington, USA)	Pupae with ecdysial problems; no adult eclosed
CG10738 RNAI-R1(NIG, Japan) [1]	Pupae with ecdysial problems; no adult eclosed
CG10738 RNAI-R2 (NIG, Japan)	Normal ecdyses

[1] Strongest line; used for results reported here.
