# Supplementary material for: Characterization of eclosion hormone receptor function reveals differential hormonal control of ecdysis during Drosophila development
Source: PLoS Genet. 2025 Aug 20;21(8):e1011672. doi: 10.1371/journal.pgen.1011672 (PMC12393706; doi:10.1371/journal.pgen.1011672)
Supplement: S3 Table — (DOCX) [file pgen.1011672.s008.docx]

**S3 Table: Summary of statistical analyses**

| **Fig.** | **Test** | **Conditions Analyzed** | **P value** | **P < 0.05?** |
| --- | --- | --- | --- | --- |
| 1E | Fisher's exact test | *w^1118^* control *vs w^1118^* +EH intensity 0 | 0.2647 | No |
| 1E | Fisher's exact test | *w^1118^* control *vs w^1118^* +EH intensity 1 | <0.0001 | Yes |
| 1E | Fisher's exact test | *w^1118^* control *vs w^1118^* +EH intensity 2 | 0.4645 | No |
| 1E | Fisher's exact test | *w^1118^* control *vs w^1118^* +EH intensity 3 | <0.0001 | Yes |
| 1E | Fisher's exact test | *w^1118^* control *vs Mi{EHR}/Df(3)EHR* + EH intensity 0 | N/A | N/A |
| 1E | Fisher's exact test | *w^1118^* control *vs Mi{EHR}/Df(3)EHR* + EH intensity 1 | 1 | No |
| 1E | Fisher's exact test | *w^1118^* control *vs Mi{EHR}/Df(3)EHR* + EH intensity 2 | 0.4668 | No |
| 1E | Fisher's exact test | *w^1118^* control *vs Mi{EHR}/Df(3)EHR* + EH intensity 3 | 0.3522 | No |
| 1E | Fisher's exact test | *w^1118^* control *vs EHR*-GAL4*/Df(3)EHR* + EH intensity 0 | N/A | N/A |
| 1E | Fisher's exact test | *w^1118^* control *vs EHR*-GAL4*/Df(3)EHR* + EH intensity 1 | 0.5145 | No |
| 1E | Fisher's exact test | *w^1118^* control *vs EHR*-GAL4*/Df(3)EHR* + EH intensity 2 | 0.3026 | No |
| 1E | Fisher's exact test | *w^1118^* control *vs EHR*-GAL4*/Df(3)EHR* + EH intensity 3 | 0.1817 | No |
| 1E | Fisher's exact test | *w^1118^* +EH *vs Mi{EHR}/Df(3)EHR* + EH intensity 0 | 0.5504 | No |
| 1E | Fisher's exact test | *w^1118^* +EH *vs Mi{EHR}/Df(3)EHR* + EH intensity 1 | <0.0001 | Yes |
| 1E | Fisher's exact test | *w^1118^* +EH *vs Mi{EHR}/Df(3)EHR* + EH intensity 2 | 0.1327 | No |
| 1E | Fisher's exact test | *w^1118^* +EH *vs Mi{EHR}/Df(3)EHR* + EH intensity 3 | <0.0001 | Yes |
| 1E | Fisher's exact test | *w^1118^* +EH vs *EHR*-GAL4*/Df(3)EHR* + EH intensity 0 | 0.5486 | No |
| 1E | Fisher's exact test | *w^1118^* +EH vs *EHR*-GAL4*/Df(3)EHR* + EH intensity 1 | <0.0001 | Yes |
| 1E | Fisher's exact test | *w^1118^* +EH vs *EHR*-GAL4*/Df(3)EHR* + EH intensity 2 | 0.08136 | No |
| 1E | Fisher's exact test | *w^1118^* +EH vs *EHR*-GAL4*/Df(3)EHR* + EH intensity 3 | <0.0001 | Yes |
| 1E | Fisher's exact test | *Mi{EHR}/Df(3)EHR* + EH vs *EHR*-GAL4*/Df(3)EHR* + EH intensity 0 | N/A | N/A |
| 1E | Fisher's exact test | *Mi{EHR}/Df(3)EHR* + EH vs *EHR*-GAL4*/Df(3)EHR* + EH intensity 1 | 0.4821 | No |
| 1E | Fisher's exact test | *Mi{EHR}/Df(3)EHR* + EH vs *EHR*-GAL4*/Df(3)EHR* + EH intensity 2 | 1 | No |
| 1E | Fisher's exact test | *Mi{EHR}/Df(3)EHR* + EH vs *EHR*-GAL4*/Df(3)EHR* + EH intensity 3 | 0.6642 | No |
|  |  |  |  |  |
| 1F | Fisher's exact test | *w^1118^* dVP+30 vs *Mi{EHR}/Df(3)EHR* dVP +2h intensity 0 | 0.06912 | No |
| 1F | Fisher's exact test | *w^1118^* dVP+30 vs *Mi{EHR}/Df(3)EHR* dVP +2h intensity 1 | <0.0001 | Yes |
| 1F | Fisher's exact test | *w^1118^* dVP+30 vs *Mi{EHR}/Df(3)EHR* dVP +2h intensity 2 | 0.004312 | Yes |
| 1F | Fisher's exact test | *w^1118^* dVP+30 vs *Mi{EHR}/Df(3)EHR* dVP +2h intensity 3 | <0.0001 | Yes |
| 1F | Fisher's exact test | *w^1118^* dVP+30 vs *EHR*-GAL4*/Df(3)EHR* dVP +2h intensity 0 | 0.1106 | No |
| 1F | Fisher's exact test | *w^1118^* dVP+30 vs *EHR*-GAL4*/Df(3)EHR* dVP +2h intensity 1 | <0.0001 | Yes |
| 1F | Fisher's exact test | *w^1118^* dVP+30 vs *EHR*-GAL4*/Df(3)EHR* dVP +2h intensity 2 | <0.0001 | Yes |
| 1F | Fisher's exact test | *w^1118^* dVP+30 vs *EHR*-GAL4*/Df(3)EHR* dVP +2h intensity 3 | <0.0001 | Yes |
| 1F | Fisher's exact test | *Mi{EHR}/Df(3)EHR* dVP +2h vs *EHR*-GAL4*/Df(3)EHR* dVP +2h intensity 0 | 1 | No |
| 1F | Fisher's exact test | *Mi{EHR}/Df(3)EHR* dVP +2h vs *EHR*-GAL4*/Df(3)EHR* dVP +2h intensity 1 | 0.1352 | No |
| 1F | Fisher's exact test | *Mi{EHR}/Df(3)EHR* dVP +2h vs *EHR*-GAL4*/Df(3)EHR* dVP +2h intensity 2 | 0.1308 | No |
| 1F | Fisher's exact test | *Mi{EHR}/Df(3)EHR* dVP +2h vs *EHR*-GAL4*/Df(3)EHR* dVP +2h intensity 0 | 0.005699 | Yes |
|  |  |  |  |  |
| 2A | Unpaired t test | *w^1118^ vs Df(3)EHR/Mi{EHR} -* Locomotion pre-TC | <0.0001 | Yes |
| 2A | Unpaired t test | *w^1118^ vs Df(3)EHR/Mi{EHR} -* Locomotion after-TC | 0.0012 | Yes |
| 2A | Unpaired t test | *w^1118^ vs Df(3)EHR/Mi{EHR} -* pre-ecdysis | N/A | N/A |
| 2A | Unpaired t test | *w^1118^ vs Df(3)EHR/Mi{EHR} -* ecdysis | 0.0289 | Yes |
| 2A | Unpaired t test | *w^1118^ vs Df(3)EHR/EHR*-GAL4 *-* Locomotion pre-TC | <0.0001 | Yes |
| 2A | Unpaired t test | *w^1118^ vs Df(3)EHR/EHR*-GAL4 *-* Locomotion after TC | 0.0187 | Yes |
| 2A | Unpaired t test | *w^1118^ vs Df(3)EHR/EHR*-GAL4 *-* pre-ecdysis | N/A | N/A |
| 2A | Unpaired t test | *w^1118^ vs Df(3)EHR/EHR*-GAL4 - ecdysis | <0.0001 | Yes |
| 2A | Unpaired t test | *w^1118^ vs UAS-EHR;EHR-*GAL4*>Df(3)EHR -* Locomotion pre-TC | 0.6559 | No |
| 2A | Unpaired t test | *w^1118^ vs UAS-EHR;EHR-*GAL4*>Df(3)EHR -* Locomotion after TC | 0.949 | No |
| 2A | Unpaired t test | *w^1118^ vs UAS-EHR;EHR-*GAL4*>Df(3)EHR -* pre-ecdysis | 0.0016 | Yes |
| 2A | Unpaired t test | *w^1118^ vs UAS-EHR;EHR-*GAL4*>Df(3)EHR -* ecdysis | 0.0445 | No |
| 2A | Unpaired t test | *Df(3)EHR/Mi{EHR} vs Df(3)EHR/EHR-*GAL4 *-* Locomotion pre-TC | 0.153 | No |
| 2A | Unpaired t test | *Df(3)EHR/Mi{EHR} vs Df(3)EHR/EHR-*GAL4 *-* Locomotion after TC | 0.2423 | No |
| 2A | Unpaired t test | *Df(3)EHR/Mi{EHR} vs Df(3)EHR/EHR-*GAL4 *-* pre-ecdysis | N/A | N/A |
| 2A | Unpaired t test | *Df(3)EHR/Mi{EHR} vs Df(3)EHR/EHR-*GAL4 *-* ecdysis | 0.0666 | No |
| 2A | Unpaired t test | *Df(3)EHR/Mi{EHR} vs UAS-EHR;EHR-*GAL4*>Df(3)EHR -* Locomotion pre-TC | <0.0001 | Yes |
| 2A | Unpaired t test | *Df(3)EHR/Mi{EHR} vs UAS-EHR;EHR-*GAL4*>Df(3)EHR -* Locomotion after TC | <0.0001 | Yes |
| 2A | Unpaired t test | *Df(3)EHR/Mi{EHR} vs UAS-EHR;EHR-*GAL4*>Df(3)EHR -* pre-ecdysis | N/A | N/A |
| 2A | Unpaired t test | *Df(3)EHR/Mi{EHR} vs UAS-EHR;EHR-*GAL4*>Df(3)EHR -* ecdysis | 0.0005 | Yes |
| 2A | Unpaired t test | *Df(3)EHR/EHR-*GAL4 *vs UAS-EHR;EHR-*GAL4*>Df(3)EHR -* Locomotion pre-TC | <0.0001 | Yes |
| 2A | Unpaired t test | *Df(3)EHR/EHR-*GAL4 *vs UAS-EHR;EHR-*GAL4*>Df(3)EHR -* Locomotion after TC | 0.0005 | Yes |
| 2A | Unpaired t test | *Df(3)EHR/EHR-*GAL4 *vs UAS-EHR;EHR-*GAL4*>Df(3)EHR -* pre-ecdysis | N/A | N/A |
| 2A | Unpaired t test | *Df(3)EHR/EHR-*GAL4 *vs UAS-EHR;EHR-*GAL4*>Df(3)EHR -* ecdysis | <0.0001 | Yes |
|  |  |  |  |  |
| 2C | Fisher's exact test | *w^1118^ vs Df(3)EHR/Mi{EHR}* | 0.4909 | No |
| 2C | Fisher's exact test | *w^1118^ vs Df(3)EHR/EHR*-GAL4 | 1 | No |
| 2C | Fisher's exact test | *w^1118^ vs UAS-EHR;EHR-*GAL4*>Df(3)EHR* | 1 | No |
| 2C | Fisher's exact test | *Df(3)EHR/Mi{EHR} vs Df(3)EHR/EHR-*GAL4 | 1 | No |
| 2C | Fisher's exact test | *Df(3)EHR/Mi{EHR} vs UAS-EHR;EHR-*GAL4*>Df(3)EHR* | 0.5368 | No |
| 2C | Fisher's exact test | *Df(3)EHR/EHR-*GAL4 *vs UAS-EHR;EHR-*GAL4*>Df(3)EHR* | 1 | No |
|  |  |  |  |  |
| 2D | One-way ANOVA + Tukey's | *w^1118^* vs. *Mi{EHR}/Df(3)EHR -* Duration of tracheal air filling | <0.0001 | Yes |
| 2D | One-way ANOVA + Tukey's | *w^1118^* vs. *EHR-*GAL4*/Df(3)EHR -* Duration of tracheal air filling | <0.0001 | Yes |
| 2D | One-way ANOVA + Tukey's | *w^1118^* vs. *UAS-EHR; EHR-*GAL4*/Df(3)EHR -* Duration of tracheal air filling | 0.9972 | No |
| 2D | One-way ANOVA + Tukey's | *Mi{EHR}/Df(3)EHR* vs. *EHR-*GAL4*/Df(3)EHR -* Duration of tracheal air filling | 0.9975 | No |
| 2D | One-way ANOVA + Tukey's | *Mi{EHR}/Df(3)EHR* vs. *UAS-EHR; EHR-*GAL4*/Df(3)EHR -* Duration of tracheal air filling | <0.0001 | Yes |
| 2D | One-way ANOVA + Tukey's | *EHR-*GAL4*/Df(3)EHR* vs. *UAS-EHR; EHR-*GAL4*/Df(3)EHR -* Duration of tracheal air filling | <0.0001 | Yes |
|  |  |  |  |  |
| 2E | Fisher's exact test | *w^1118^ vs Df(3)EHR/Mi{EHR}* | <0.0001 | Yes |
| 2E | Fisher's exact test | *w^1118^ vs Df(3)EHR/EHR*-GAL4 | <0.0001 | Yes |
| 2E | Fisher's exact test | *w^1118^ vs UAS-EHR;EHR-*GAL4*>Df(3)EHR* | 0.1895 | No |
| 2E | Fisher's exact test | *Df(3)EHR/Mi{EHR} vs Df(3)EHR/EHR-*GAL4 | 0.7395 | No |
| 2E | Fisher's exact test | *Df(3)EHR/Mi{EHR} vs UAS-EHR;EHR-*GAL4*>Df(3)EHR* | 0.0002647 | Yes |
| 2E | Fisher's exact test | *Df(3)EHR/EHR-*GAL4 *vs UAS-EHR;EHR-*GAL4*>Df(3)EHR* | <0.0001 | Yes |
|  |  |  |  |  |
| 3A | Unpaired t test | *w^1118^*>*EHR-*GAL4 vs *w^1118^*>2x*Kir2.1* - Locomotion pre-TC | 0.7721 | No |
| 3A | Unpaired t test | *w^1118^*>*EHR-*GAL4 vs *w^1118^*>2x*Kir2.1* - Locomotion after TC | 0.8184 | No |
| 3A | Unpaired t test | *w^1118^*>*EHR-*GAL4 vs *w^1118^*>2x*Kir2.1* - pre-ecdysis | 0.9387 | No |
| 3A | Unpaired t test | *w^1118^*>*EHR-*GAL4 vs *w^1118^*>2x*Kir2.1* - ecdysis | 0.7045 | No |
| 3A | Unpaired t test | *w^1118^*> *EHR-*GAL4 vs *EHR-*GAL4>2x*Kir2.1* - Locomotion pre-TC | <0.0001 | Yes |
| 3A | Unpaired t test | *w^1118^*>*EHR-*GAL4 vs *EHR-*GAL4>2x*Kir2.1* - Locomotion after TC | <0.0001 | Yes |
| 3A | Unpaired t test | *w^1118^*>*EHR-*GAL4 vs *EHR-*GAL4>2x*Kir2.1* - pre-ecdysis | 0.0091 | Yes |
| 3A | Unpaired t test | *w^1118^*>*EHR-*GAL4 vs *EHR-*GAL4>2x*Kir2.1* - ecdysis | <0.0001 | Yes |
| 3A | Unpaired t test | *w^1118^*>2x*Kir2.1* vs *EHR-*GAL4>2x*Kir2.1* - Locomotion pre-TC | 0.0005 | Yes |
| 3A | Unpaired t test | *w^1118^*>2x*Kir2.1* vs *EHR-*GAL4>2x*Kir2.1* - Locomotion after TC | <0.0001 | Yes |
| 3A | Unpaired t test | *w^1118^*>2x*Kir2.1* vs *EHR-*GAL4>2x*Kir2.1* - pre-ecdysis | 0.014 | Yes |
| 3A | Unpaired t test | *w^1118^*>2x*Kir2.1* vs *EHR-*GAL4>2x*Kir2.1* - ecdysis | <0.0001 | Yes |
| 3A | Unpaired t test | *w^1118^*>*rpr* vs *EHR-*GAL4>*rpr* - Locomotion pre-TC | 0.1235 | No |
| 3A | Unpaired t test | *w^1118^*>*rpr* vs *EHR-*GAL4*>rpr* - Locomotion after TC | <0.0001 | Yes |
| 3A | Unpaired t test | *w^1118^*>*rpr* vs *EHR*-GAL4>*rpr* - pre-ecdysis | N/A | N/A |
| 3A | Unpaired t test | *w^1118^*>rpr vs *EHR-*GAL4>*rpr* - ecdysis | <0.0001 | Yes |
|  |  |  |  |  |
| 3C | Fisher's exact test | *w^1118^*>*EHR-*GAL4 vs *w^1118^*>2x*Kir2.1* | 1 | No |
| 3C | Fisher's exact test | *w^1118^*> *EHR-*GAL4 vs *EHR-*GAL4>2x*Kir2.1* | 0.0001554 | Yes |
| 3C | Fisher's exact test | *w^1118^*>*rpr* vs *EHR-*GAL4>*rpr* | 0.004662 | Yes |
|  |  |  |  |  |
| 3D | Fisher's exact test | *w^1118^*>*EHR-*GAL4 vs *w^1118^*>2x*Kir2.1* | 1 | No |
| 3D | Fisher's exact test | *w^1118^*> *EHR-*GAL4 vs *EHR-*GAL4>2x*Kir2.1* | 0.0001554 | Yes |
| 3D | Fisher's exact test | *w^1118^*>*rpr* vs *EHR-*GAL4>*rpr* | 0.004662 | Yes |
|  |  |  |  |  |
| 3E | Fisher's exact test | *w^1118^*>2x*Kir2.1 vs w^1118^*> *tub*-Gal80^ts^;*EHR-*GAL4 | 0.5547 | No |
| 3E | Fisher's exact test | *w^1118^*>2x*Kir2.1 vs tub*-Gal80^ts^;*EHR-*GAL4>2x*Kir2.1* | <0.0001 | Yes |
| 3E | Fisher's exact test | *vs w^1118^*> *tub*-Gal80^ts^;*EHR-*GAL4 *vs tub*-Gal80^ts^;*EHR-*GAL4>2x*Kir2.1* | <0.0001 | Yes |
|  |  |  |  |  |
| 3F | Fisher's exact test | *w^1118^*>2x*Kir2.1 vs w^1118^*> *tub*-Gal80^ts^;*EHR-*GAL4 | 0.8413 | No |
| 3F | Fisher's exact test | *w^1118^*>2x*Kir2.1 vs tub*-Gal80^ts^;*EHR-*GAL4>2x*Kir2.1* | <0.0001 | Yes |
| 3F | Fisher's exact test | *vs w^1118^*> *tub*-Gal80^ts^;*EHR-*GAL4 *vs tub*-Gal80^ts^;*EHR-*GAL4>2x*Kir2.1* | <0.0001 | Yes |
|  |  |  |  |  |
| 7A | Fisher's exact test | EHR[-] average vs UAS-*EHR*; *EHR*-GAL4/*Df(3)EHR* | 0 | Yes |
| 7A | Fisher's exact test | EHR[-] average vs *CCAP>EHR; Mi{EHR}/Df(3)EHR* | 0 | Yes |
| 7A | Fisher's exact test | EHR[-] average vs *ETH>EHR; Mi{EHR}/Df(3)EHR* | 0.0331 | Yes |
| 7A | Fisher's exact test | EHR[-] average vs *EHups>EHR; Mi{EHR}/Df(3)EHR* | 0.8238 | No |
| 7A | Fisher's exact test | EHR[-] average vs *VGlut>EHR; Mi{EHR}/Df(3)EHR* | 0.004 | Yes |
| 7A | Fisher's exact test | EHR[-] average vs UAS-*EHR; 386Y, Mi{EHR}/Df(3)EHR* | 0 | Yes |
| 7A | Fisher's exact test | EHR[-] average vs *C929>EHR; Mi{EHR}/Df(3)EHR* | 0 | Yes |
| 7A | Fisher's exact test | EHR[-] average vs *rk>EHR; Mi{EHR}/Df(3)EHR* | 0.3432 | No |
| 7A | Fisher's exact test | EHR[-] average vs *btl>EHR; Mi{EHR}/Df(3)EHR* | 0 | Yes |
|  |  |  |  |  |
| 7B | Fisher's exact test | EHR[-] average vs UAS-*EHR; EHR*-GAL4/*Df(3)EHR* | 0 | Yes |
| 7B | Fisher's exact test | EHR[-] average vs *CCAP>EHR; Mi{EHR}/Df(3)EHR* | 0.0154 | Yes |
| 7B | Fisher's exact test | EHR[-] average vs *ETH>EHR; Mi{EHR}/Df(3)EHR* | 0 | Yes |
| 7B | Fisher's exact test | EHR[-] average vs *EHups>EHR; Mi{EHR}/Df(3)EHR* | 0.001 | Yes |
| 7B | Fisher's exact test | *EHR[-] average vs VGlut>EHR; Mi{EHR}/Df(3)EHR* | *1* | No |
| 7B | Fisher's exact test | EHR[-] average vs UAS-*EHR; 386Y, Mi{EHR}/Df(3)EHR* | 0 | Yes |
| 7B | Fisher's exact test | EHR[-] average vs *C929>EHR; Mi{EHR}/Df(3)EHR* | 0 | Yes |
| 7B | Fisher's exact test | EHR[-] average vs *rk>EHR; Mi{EHR}/Df(3)EHR* | 0.1067 | No |
| 7B | Fisher's exact test | EHR[-] average vs *btl>EHR; Mi{EHR}/Df(3)EHR* | 0.0024 | Yes |
|  |  |  |  |  |
| 7C | Fisher's exact test | EHR[-] average vs UAS-*EHR; EHR-GAL4/Df(3)EHR* | 0 | Yes |
| 7C | Fisher's exact test | EHR[-] average vs *CCAP>EHR; Mi{EHR}/Df(3)EHR* | 0.1924 | No |
| 7C | Fisher's exact test | EHR[-] average vs *ETH>EHR; Mi{EHR}/Df(3)EHR* | 1 | No |
| 7C | Fisher's exact test | EHR[-] average vs *EHups>EHR; Mi{EHR}/Df(3)EHR* | 1 | No |
| 7C | Fisher's exact test | EHR[-] average vs *VGlut>EHR; Mi{EHR}/Df(3)EHR* | 1 | No |
| 7C | Fisher's exact test | EHR[-] average vs UAS-*EHR; 386Y, Mi{EHR}/Df(3)EHR* | 0 | Yes |
| 7C | Fisher's exact test | EHR[-] average vs *C929>EHR; Mi{EHR}/Df(3)EHR* | 0.0374 | Yes |
| 7C | Fisher's exact test | EHR[-] average vs *rk>EHR; Mi{EHR}/Df(3)EHR* | 0.2697 | No |
| 7C | Fisher's exact test | EHR[-] average vs *btl>EHR; Mi{EHR}/Df(3)EHR* | 0.1376 | No |
|  |  |  |  |  |
| 7D | Fisher's exact test | EHR[-] average vs UAS-*EHR; EHR-GAL4/Df(3)EHR* | N/A | N/A |
| 7D | Fisher's exact test | EHR[-] average vs *CCAP>EHR; Mi{EHR}/Df(3)EHR* | N/A | N/A |
| 7D | Fisher's exact test | EHR[-] average vs *ETH>EHR; Mi{EHR}/Df(3)EHR* | N/A | N/A |
| 7D | Fisher's exact test | EHR[-] average vs *EHups>EHR; Mi{EHR}/Df(3)EHR* | N/A | N/A |
| 7D | Fisher's exact test | EHR[-] average vs *VGlut>EHR; Mi{EHR}/Df(3)EHR* | N/A | N/A |
| 7D | Fisher's exact test | EHR[-] average vs UAS-*EHR; 386Y, Mi{EHR}/Df(3)EHR* | N/A | N/A |
| 7D | Fisher's exact test | EHR[-] average vs *C929>EHR; Mi{EHR}/Df(3)EHR* | N/A | N/A |
| 7D | Fisher's exact test | EHR[-] average vs *rk>EHR; Mi{EHR}/Df(3)EHR* | N/A | N/A |
| 7D | Fisher's exact test | EHR[-] average vs *btl>EHR; Mi{EHR}/Df(3)EHR* | N/A | N/A |
|  |  |  |  |  |
| 8E | LME model | Rest population mean time to peak | 0.052509 | No |
| 8E | LME model | CCAP Left population mean time to peak | 0.037704 | Yes |
| 8E | LME model | CCAP Right population mean time to peak | 0.1723 | No |
| 8E | LME model | Central Right population mean time to peak | 0.83524 | No |
| 8E | LME model | Central Left population mean time to peak | 0.84542 | No |
| 8E | LME model | Posterior Right population mean time to peak | 0.71989 | No |
|  |  |  |  |  |
| 8F | Bonferroni + Wilcoxon rank | Rest population vs CCAP Left population | 0.438837 | No |
| 8F | Bonferroni + Wilcoxon rank | Rest population vs CCAP Right population | 0.378358 | No |
| 8F | Bonferroni + Wilcoxon rank | Rest population vs Central Left population | 1.000000 | No |
| 8F | Bonferroni + Wilcoxon rank | Rest population vs Central Right population | 0.671896 | No |
| 8F | Bonferroni + Wilcoxon rank | Rest population vs Posterior (POS) population | 1.000000 | No |
| 8F | Bonferroni + Wilcoxon rank | CCAP Left population vs CCAP Right population | 1.000000 | No |
| 8F | Bonferroni + Wilcoxon rank | CCAP Left population vs Central Right population | 0.007175 | Yes |
| 8F | Bonferroni + Wilcoxon rank | CCAP Left population vs Central Left population | 0.000217 | Yes |
| 8F | Bonferroni + Wilcoxon rank | CCAP Left population vs Posterior (POS) population | 0.021207 | Yes |
| 8F | Bonferroni + Wilcoxon rank | CCAP Right population vs Central Left population | 0.025342 | Yes |
| 8F | Bonferroni + Wilcoxon rank | CCAP Right population vs Central Right population | 0.000517 | Yes |
| 8F | Bonferroni + Wilcoxon rank | CCAP Right population vs Posterior (POS) population | 0.020122 | Yes |
| 8F | Bonferroni + Wilcoxon rank | Central Left population vs Central Right population | 1.000000 | No |
| 8F | Bonferroni + Wilcoxon rank | Central Left population vs Posterior (POS) population | 1.000000 | No |
| 8F | Bonferroni + Wilcoxon rank | Central Right population vs Posterior (POS) population | 1.000000 | No |
|  |  |  |  |  |
| S2A | Fisher's exact test | *w^1118^*>UAS-*TrpA1* vs *w^1118^*>*EHR*-GAL4 | 1 | No |
| S2A | Fisher's exact test | *w^1118^*>UAS-*TrpA1* vs *EHR*-GAL4> UAS-*TrpA1* | 0.0000258 | Yes |
| S2A | Fisher's exact test | *w^1118^*>*EHR*-GAL4 vs *EHR*-GAL4> UAS-*TrpA1* | 0.000003127 | Yes |
| S2B | Fisher's exact test | *w^1118^*>UAS-*TrpA1* vs *w^1118^*>*EHR*-GAL4 | 0.2322 | No |
| S2B | Fisher's exact test | *w^1118^*>UAS-*TrpA1* vs *EHR*-GAL4> UAS-*TrpA1* | <0.0001 | Yes |
| S2B | Fisher's exact test | *w^1118^*>*EHR*-GAL4 vs *EHR*-GAL4> UAS-*TrpA1* | <0.0001 | Yes |
