# Supplementary material for: Characterization of eclosion hormone receptor function reveals differential hormonal control of ecdysis during Drosophila development
Source: PLoS Genet. 2025 Aug 20;21(8):e1011672. doi: 10.1371/journal.pgen.1011672 (PMC12393706; doi:10.1371/journal.pgen.1011672)
Supplement: Attachment — Submitted filename: Response_to_reviewers.pdf [file pgen.1011672.s010.pdf]

CENTRO INTERDISCIPLINARIO DE
Neurociencia de
Valparaíso

Universidad
de Valparaíso
CHILE
Instituto de Neurociencia
Facultad de Ciencias

June 22nd, 2025

Editor
PLoS Genetics

Dear PLoS Genetics Editor,

We were very pleased by the positive reception of our work. Below we have responded to each point raised by the reviewers. Changes made to the manuscript have been indicated using red font in the version called "Marked Revised Manuscript".

We certify that we are the authors of all the photos included in this manuscript (Figures 1A-D, 2B, 3B, 4B-E, 5, 6, S3, and S4).

We hope that our work is now suitable for publication in PLoS Genetics.

Best wishes,

John Ewer, Ph.D.
Instituto de Neurociencia, Facultad de Ciencias,
Universidad de Valparaíso, Valparaíso, Chile
email: john.ewer@uv.cl
ewerlab.org

Signed on behalf of all authors: Valeria Silva, Robert Scott, Paulina Guajardo, Haojiang Luan, Ruben Herzog, and Benjamin White.

We were very pleased by the positive reception of our work. Below we have responded to each point raised by the reviewers. Changes made to the manuscript have been indicated using red font.

Reviewer #1:

This is an exceptionally clear and incisive report on the neuropeptide EH receptor in Drosophila. EH (eclosion hormone) is part of a complex system underlying neuropeptide-regulated behavior, that features hierarchical control levels, and mutual positive feedback between the two key excitatory neuropeptides at the top of the hierarchy (ETH and EH). The regulated behavior is ecdysis, which represents the final step in the molting process of insects whereby the newly molted animal breaks out of its old cuticle through a set of stereotyped behaviors. The behavioral sequence promotes extraction of the body and appendages without damage and occurs at the end of each molt; embryonic and adult ecdysis are termed eclosion. The EHR had been a poorly understood element in the system. This report provides much-needed information on its expression and functional utilization in diverse target cells. Unlike the majority of neuropeptide receptors, EHR is a membrane guanylate cyclase which adds novelty to the report. Other strengths found in the paper – they perform extensive mosaic analysis using split Gal4s to map the identities of the many EHR-expressing cells. They also perform mosaic EHR expression to relate different target cells to different stage-specific behaviors. They also document Ca dynamics in EHR cells of the larval CNS following exposure to EH. In all, this is an exciting, technically-proficient and highly-informative paper.

We were very happy to receive such positive comments on our work!

Minor concerns

Figure 1. The intensity of anti-ETH immunoreactivity is quantified as a function of time in Inka cells. Quantification is presented as arbitrary subjective categories. Is it possible to measure fluorescence intensity?
Although we do have images of every Inka cell that was scored, we opted to score the staining qualitatively rather than quantitatively because it is much faster and pilot tests showed that the results obtained are not qualitatively different. Although the reviewer is correct that the scoring used is subjective, it was done blind to both genotype and condition, and has been our method of choice in a number of publications (e.g., Clark et al., 2004; Lahr et al, 2012; Kruger et al., 2015; refs 36, 53 and 28, respectively). Further details of the quantification methods were added to the Methods section (line 648). And as requested by Reviewer 2, we analyzed the statistical significance of these results, which is summarized in the legend to Figure 1 and shown in Table S3.

Figure 2. In the EHR mutant state, the locomotion that precedes the TC stage appears to start early and its duration appears longer due to the premature onset. Alternatively, could it start at the correct time but extend in duration because the TC stage is delayed? Are there independent markers that suggest whether the behavior of the TC is anomalously displayed?
The reviewer is indeed correct that there appear to be some differences in the duration of the locomotion period prior to TC. Unfortunately we have no markers to distinguish between the two alternatives suggested. In addition, these differences may not be entirely real. Indeed, in the mutants the different phases of ecdysis are much less discrete than they are in the

controls, so an extended locomotory phase may really reflect a much slower and less well-defined time of onset of tracheal collapse.

Figure 4A. Suggest placing the pink Inka cells in the schematic on the side of the trachea, for better visibility.
Thank you for the suggestion. We have now done so.

Figure 4 B/C. Why are the majority of EHR-nls RFP cells not labeled by EHR> mCD8? Only the Inka cells appear to be mCD8-+. Also, the Legend says the Inka cells is readily visible with nuclear reporter in 1C – but that is not visible to me.
The reviewer is correct in their appreciation of these figures. The problem of showing EHR expression in Inka cells and in tracheal cells using the same reporter has, in fact, been very vexing. Indeed, we found that driving a membrane-bound GFP with EHR-GAL4 labeled the Inka cells but the tracheal cells were barely visible; and the converse occurred using a nuclear RFP. We don't know the reasons for this. Maybe the tracheal cells are (comparatively) so big that the membrane GFP signal becomes diluted and is therefore much weaker than that of the Inka cells; and maybe the nuclear signal reflects the developmental expression of EHR, which would be expected to be low in the 3rd instar larvae used for these preparations. Regardless of the reason, we revised Figure 4B and a) now provide a better picture of the nuclear RFP signal and b) eliminated the panels that used a membrane-bound GFP.

Figure 5. EHR in larval CNS at the 3rd instar. How does this compare to the 1st instar CNS? Is there an increase in cell number?
This is a good point and is one we have not explored. However, we would not expect the pattern of EHR expression to change between these 2 stages because their corresponding ecdysis behavior is indistinguishable (apart from the differences in their duration).

Are there differences in ecdysis behavior between the one at 1 to 2, versus 3 to Pupal?
As mentioned above, ecdysis behavior from the first to the second larval instar is indistinguishable from ecdysis behavior from the second to the third larval instar, except that the latter is longer in duration. By contrast, pupal ecdysis is a much more complex behavior, which involves not only ecdysis itself but also head eversion and imaginal disc eversion and extension. Based on the results summarized in Figure 8 and Table 2, pupation was a much more difficult behavior to rescue, which could suggest that it requires EHR function in a larger complement of cells and neurons.

Figure 6. EHR expression in the pharate adult CNS. Is this pattern retained in the adult? Or do these cells die are their use at adult eclosion?
We have not investigated this question. Some neurons are known to die (Vm EH neurons, some CCAP neurons, etc) but others are known to persist (e.g., Inka cells). For most neurons their fate as well as whether they express EHR is unknown. Answering this question rigorously would probably require using *in situ* hybridization because, in our experience, perdurance of reporter expression (e.g., GFP) can produce labeling in cells that no longer express the relevant receptor. More importantly, expression of GAL4, which would be used to drive the expression of any reporter used, can interfere with the normal timing of programmed cell death. Because of these 2 issues we have not addressed this question rigorously; in the context of the current study we also consider that it does not contribute critical information since adult emergence is the final ecdysis in *Drosophila*.

Figure 8. The major lines are marked by dark versus light grey – these are very similar and I suggest you try something else.
We appreciate this comment especially since we agree that this figure was indeed difficult to follow. We have now revised it and incorporated a color scheme which we hope will help visualize the results.

Given expression of EHR in Vm cells it may be worth noting whether ETH-R is expressed by Inka cells.
This is a good point, as it could suggest that ETH is also involved in ETH release. We have examined this question and found that ETHR is expressed in a cell type that sits immediately adjacent to, but is clearly distinct from, the Inka cells. We have added this information to the manuscript (lines 258-259) a supplementary figure to illustrate this point (Fig. S4).

Is EHR also expressed by other EH-expressing neurons (non-Vm)?
Yes, it is, in the Dl EH neurons (see Figure 6E)

Also, EH-Gal4 is not marked when used.
We are not quite sure what this comment refers to but we may have created some confusion by not being consistent in our naming the 2 GAL4 drivers that we used to drive expression in EH-expressing cells (EHups-GAL4 and Ehpan-GAL4). We have now made this naming more consistent and hope that the concern has been resolved.

Also UAS-EHR is only marked when used in the case of EH-Gal4, but not when used with other Gal4s.
We apologize for the slight inconsistencies in our nomenclatures but it was to avoid confusions. For instance, when we write EHR>EHR it is clear that we are referring to EHR-GAL4 driving expression of UAS-EHR. However, when EHR is mentioned alone (as occurs for some control genotypes) then just writing “EHR" would create some ambiguity, as it wouldn’t be clear if we are referring to EHR-GAL4 or to UAS-EHR alone. In such cases we decided it was better to spell out the genotype.

Reviewer #2:
EH is an important regulatory neuropeptide involved in insect molting or ecdysis. The EH receptor (EHR) has been identified in a few other insect species, including the oriental fruit fly and the desert locust; however, its function in Drosophila melanogaster, a model insect for ecdysis research, remains unexplored. In this study, the authors investigated the role of EHR in fruit fly ecdysis using newly developed molecular genetic tools, including EHR mutant and Gal4 lines. Comprehensive molecular genetic analyses using targeted knockdown and genetic rescue experiments revealed that the EHR's role varies depending on the developmental stage and target. This work is an important contribution to resolving the long-standing uncertainty about the neuronal functions of EH in Drosophila ecdysis. However, Figure 7 is not sufficiently developed and, in its current form, is not relevant to the functions of EHR that are the focus of this manuscript.

Major comments,
In Figure 7, the authors showed that ETH stimulates neural activity patterns in neurons that express EHR (most of which also express ETHR, such as CCAP neurons). The observation

that ETH induces neural activity patterns in these neurons has been reported several times, both by the authors' group and by other groups. The actual new important finding should be to investigate the role of EHR in regulating the neural activity of these neurons stimulated by ETH. In addition, co-treatment of the CNS with EH could induce changes in the fictive ecdysis patterns induced by ETH, allowing the authors to investigate the function of EHR in such changes. This is particularly important because the authors claim that EH and ETH acts synergistically in the brain neurons.
We agree with the reviewer that it would be very informative to know how the CNS responds to EH, and how ETH contributes to this response. Unfortunately, such experiments would currently be extremely challenging to do for 2 reasons. The first is because the window of sensitivity to EH is extremely narrow. For example, in the case of the larva, Inka cells did not respond to EH unless the cells were challenged less than 10 minutes before ecdysis (results shown in Figure 1). In the case of the larva, we have reliable markers that occur shortly before ecdysis; by contrast, we don't have such markers for pupal ecdysis, which is the stage when these *in vitro* experiments are done (because the CNS is largest at this stage). More importantly, it is very likely that EH does not cross the blood-brain barrier; at least in Manduca sexta, preparations must be desheathed for EH to cause a CNS response (Gammie & Truman, 1999; https://doi.org/10.1242/jeb.202.4.343). In any event, our current understanding of how EH acts within the CNS is that it is released as a result of the actions of ETH: ETH released into the hemolymph causes EH release within the CNS. And we know that EH has an important role within the CNS that goes beyond triggering/amplifying the release of ETH from Inka cells because the neuronal response to ETH is different in an Eh mutant background (Mena et al., 2016; ref 25), and because the behavioral defects of Eh mutants cannot be rescued by ETH injections (Kruger et al., 2015, ref 28). Thus, currently the only feasible way to cause EH release within the CNS *in vitro* is to stimulate it with ETH. We do, nevertheless, hope to address these important questions in future experiments, for instance by stimulating EH release optogenetically.

The authors also discovered an early activation of CCAP cells in ETH-induced fictive pupal ecdysis, compared to other EHR neurons. Since CCAP cells are also ETHR cells, the activation of CCAP cells during pupal ecdysis is expected to be directly induced by ETHR rather than EHR. Without showing the role of EHR in such CCAP cell function, the relevance of this result to this work is limited. I suggest to remove it.
We focused on the CCAP neurons because they are one of the few populations that we are relatively confident that we can identify in a preparation in which EHR drives GCaMP expression, and whose time course we know well. Thus, they serve as a reference, rather than as an interesting population as such. We have added this reasoning to the text (lines 452-453). And we know that EH contributes to the response of CCAP neurons to ETH because their response to this challenge differs significantly in an Eh mutant background (Mena *et al.*, 2016; ref 25).

EHR in CCAP neurons rescued larval ecdysis EHR mutants to some extent (Table 2). This does not fit well with the previous observation that CCAP neurons are not required for larval ecdysis in Drosophila. Does this require some explanation?
The reviewer is quite right that CCAP neurons are not essential for larval ecdysis. Nevertheless, we showed in Park *et al*. (2003; https://doi.org/10.1242/dev.00503) that the targeted ablation of CCAP neurons caused a significant lengthening of pre-ecdysis and a tripling of the duration of ecdysis itself. Thus, while not *essential*, these neurons do play a role

at ecdysis, and our results show that this role appears to be *sufficient* to partially rescue the lethality of EHR mutants.

Minor points

1. Authors claim that EHR is a bona fide receptor for EH. Thus, it would be useful to include EH mutants at least in Table 2.

We thank the reviewer for this suggestion, and have now added these results to Figure 7 and Table 2. The levels of lethality obtained in *Eh* null mutant animals were similar to those seen in animals bearing the different EHR null alleles we used. It is especially significant that all 78 animals that reached the third instar larval stage were unable to pupate and died with the signature signs of pupal ecdysial failure (no head eversion, short legs and wings, etc).

2. 64 lines. “that that”

Thank you for catching this error. It has now been corrected.

3. Fig. 2B, 3B. Include enlarged image of the mouth part.

Thank you for the suggestion. A corresponding higher magnification image has now been included in Figures 2 and 3.

4. Fig. 2A, 2C, 2E, 3A, 3C-F, 8A-D. Include statistical comparisons.

Thank you for the suggestion. We have now carried out these statistical analyses. The results are indicated in the legend to Figure 2, and within Figures 3 and 7 (formerly Figure 8). The exact values obtained are reported in Table 2. For Figure 7, comparisons were done relative to the average percentage of rescue obtained for the different EHR mutants that were used.

5. Fig. 4F. What about other imaginal discs?

We found that only the leg discs expressed EHR. Our interest in the leg imaginal discs was motivated by our previous observation that EH expression is elevated in the associated cells surrounding Keilin’s organ.

6. Table 1. What does 'ok' mean? Do all 'ok's indicate ok^3? If so, why do some genotypes have ‘ok’ labels in larval and pupal ecdysis, while others have it only in pupal ecdysis? What does ‘-’ indicate?

We apologize for the confusing label. We have now replaced “ok” and “-“ by “ND” and indicated at the foot of the table that success at this ecdysis was not specifically determined.

Reviewer #3:

This paper tests the hypothesis that the Drosophila gene CG10738, a homolog of the eclosion hormone receptor-expressing gene previously described in the oriental fruit fly Bactrocera dorsalis, encodes the receptor for eclosion hormone (EHR). Through a series of elegant experiments, the authors provide very convincing evidence that this is the case. They have shown that cells expressing EHR respond to synthetic eclosion hormone (EH), that EHR-deficiency causes lethal ecdysis phenotypes in all stages of development (larval, pupal, adult), and that expression of this receptor in EHR mutant cells rescues lethal phenotypes. Of great interest are findings that EHR and ecdysis-triggering hormone (ETH) receptors are co-expressed in cells that produce these signaling molecules (Inka cells and Vm neurons) and in many “downstream” target cells known to be involved in orchestrating the ecdysis behavioral sequence. In addition to its role in coordinating ecdysis behaviors, results support key

functional roles for EHR in tracheal airfilling, wing expansion, cibarial pumping, location in leg imaginal discs associated with Keilin's organ and in other adult structures. EHR-expressing cells are mapped in the central nervous system and patterns of cellular activity during fictive pupal ecdysis have been monitored via calcium imaging.

This study of eclosion hormone functions is a tour de force that demonstrates how a behavioral sequence is programmed by a feedback and feed-forward modulatory signaling network. The work builds upon and refines previous studies by the authors and others, and greatly expands our understanding of how a complex behavior is initiated and scheduled.

We were very happy to receive such positive comments on our work from an expert in this field.

Specific comments

1. There is very little to criticize about this work. A few suggestions are offered in the interests of promoting clarity.
These suggestions are much appreciated!

2. Synthetic eclosion hormone. How was it purified from the cell lysate? Presumably nickel resin. Were the signal sequence and His-tag portions cleaved from the mature peptide? If not, it is interesting that the entire peptide retained activity. Did disulfide bond formation occur prior to cell lysis? Within the Sf9-expressing cells? How was concentration determined?
The synthesis of EH was a rather frustrating process. Synthesis and purification were carried out by GenScript (Hong Kong) using the pFastBac baculovirus. Unfortunately, the product was never secreted, despite trying different signal peptides. At the end, the protein was purified from a cell lysate. Its purity was determined by Western analysis and its concentration using a Bradford assay. The status of the signal peptide and whether it was correctly folded was not determined. These details have now been added to the Methods section (lines 625-631).

3. Fig. 2. Please explain in more detail the terms "locomotion pre TC" and "locomotion after TC" in Fig. 2A. Although this is somewhat explained in the Methods, perhaps it could be included in the figure legend. Are they simply normal crawling movements?
Yes, "locomotion" simply refers to crawling. This has now been indicated in the legend to Figure 2, as suggested.

4. The cells that produce ETH are variously referred to as Inka cells, ETH cells, epitracheal cells. It would be helpful to settle upon a consistent name (Inka cells?), especially since they are not a heterogenous population of cells.
We agree that the various ways we refer to the Inka cells may be confusing. We now refer to them only as Inka cells (and in introductory sections as "epitracheal Inka cells")

5. The three sentences beginning with line 177 could be re-ordered or re-formulated. It is stated that premature activation of EHR-expressing cells causes 100% lethality and therefore these cells are essential for ecdysis success. The third sentence alludes to the necessity of correct timing, but this may not be so clear to the uninitiated reader.
Thank you for pointing this out. Furthermore, considering that we never tested the consequences of killing, inactivating, or activating EHR-expressing cells at *different times*, we can't really say that our results "… also suggest that the timing and the sequence of activation

of EH targets is critical for the proper orchestration of ecdysis.". Thus, we opted to simply delete the sentence that was originally on lines 180-182.

6. Line 214. Control of ecdysis by EH through both feed-forward and feedback mechanisms fits with the previous work of Diao et al. (Ref 17) on neuromodulatory connectivity. It is surprising that this is not brought out in the Discussion section.
Thank you for pointing this out! We have now added the following text (see lines 481-499). "EH signaling to neurons that express CCAP and bursicon, as well as to downstream neurons targeted by these two peptides, demonstrates the existence of feedforward loop that overlaps with the one previously described for ETH (Kim *et al.*, 2006; Diao *et al.*, 2016; refs 16 and 26). The latter loop includes the EH-expressing Vm neurons and the CCAP/bursicon neurons, and our results here indicate the presence of other possible loops formed with additional neurons that are jointly targeted by EH and ETH. These data indicate that the neuromodulatory networks that control ecdysis include coupled feedforward loops organized in a hierarchical manner, consistent with the model proposed earlier by Diao *et al.* (2017; ref 17). Sequential execution of ecdysis behavior is presumably determined by the progression of activity through these loops, which also incorporate feedback control, mediated at least in part by EH. In addition to the positive feedback loop formed by EH and ETH signaling, our results suggest an additional possible autocrine loop in which the Vm neurons respond to their own release of EH. However, some caution is required in interpreting this finding since it is possible that the Vms are responding to EH released by the small subset of recently discovered "non-canonical" EH-expressing neurons, which are not targets of ETH (Scott et al., 2020; ref 29). In general, however, the topology of neuromodulatory connections among ecdysis neurons suggests broadly distributed, coordinated control of behavior in which feedforward loops support staggered, progressive changes in motor program output."

7. Lines 339-341: This sentence states that because small subsets of EHR-expressing neurons failed to rescue ecdysial defects, it is concluded that many EH targets are important for ecdysis success. The logic here is difficult to discern.
The only driver that caused significant rescue of all ecdyses was C929, which is expressed in many neurons and cells. We agree that it remains possible that we simply failed to target a specific small population of EHR-expressing neurons that could mediate rescue. However, we favor the idea of EH action being distributed over multiple targets. This explanation is consistent with the broad expression pattern of EHR and the fact that expression needs to be restored in much of this pattern to obtain rescue. We have added this argument to support our tentative interpretation to lines 506-509.

8. Line 500: Please define dMH stage of 1st instar larvae (spell it out for the non-initiated).
Thank you; this detail has been added (line 635).

9. Line 538: Was this Drosophila ETH1?
Thank you for making this point; yes, it was ETH1. This information has now been added to the Methods (line 711)

10. Regarding the Supplementary video, is it not possible to label/identify at least some of the EHR neurons responding to ETH?

Thank you for the suggestion, Examples of the relevant classes of neurons (“Posterior”, “Central Right”, “Central Left”, “CCAP Right” and “CCAP Left”) have now been labeled in the video.
