# Supplementary material for: Characterization of eclosion hormone receptor function reveals differential hormonal control of ecdysis during Drosophila development
Source: PLoS Genet. 2025 Aug 20;21(8):e1011672. doi: 10.1371/journal.pgen.1011672 (PMC12393706; doi:10.1371/journal.pgen.1011672)
Supplement: Attachment — Submitted filename: Response_2.pdf [file pgen.1011672.s011.pdf]

August 1st, 2025

Editor
PLoS Genetics

Dear PLoS Genetics Editor,

We were very pleased by the positive reception of our revised work. Below we have responded to the additional points raised by the reviewers. Changes made to the manuscript have been indicated using red font in the marked version of the manuscript.

We certify that we are the authors of all the photos included in this manuscript (Figures 1A-D, 2B, 3B, 4B-E, 5, 6, S3, and S4).

We hope that our work is now suitable for publication in PLoS Genetics. May I add that one of the authors faces their annual review soon. Thus, we would be very grateful if a final verdict on our manuscript could be made soon.

Best wishes and many thanks for your consideration.

John Ewer, Ph.D.
Instituto de Neurociencia, Facultad de Ciencias,
Universidad de Valparaíso, Valparaíso, Chile
email: john.ewer@uv.cl
ewerlab.org

Signed on behalf of all authors: Valeria Silva, Robert Scott, Paulina Guajardo, Haojiang Luan, Ruben Herzog, and Benjamin White.

Response to reviewers

Reviewer #1:

The authors did an excellent job in responding to reviewer's concerns and questions, including mine. With a second opportunity to review the findings, I had a couple of additional questions for minor concerns.

First on a technical note, I see that Flybase predicts two different protein isoforms for CG10738 (EHR). They differ by about 15 amino acids in the middle of the protein. Is there any information about this from prior studies of EHR? Or from other guanylate cyclases? Which form (PC/PD or PE/PF) was used in the rescue experiments? The methods section does not include that detail.

We were very happy to read that this reviewer was satisfied with our revisions.

Regarding the predicted isoforms of CG10738 (EHR) there are in fact 4: taking isoform called "C" in Flybase as the canonical sequence, isoforms D and E each include a novel short exon (between exons 7 and 8) that encodes 2 additional amino acids (HQ), and isoforms E and F both have a short exon 10 that lacks coding sequence for 13 amino acids (HLLNHLPRRLPPQ). An analysis of these different isoforms shows that they all contain the same domains (i.e. the predicted ligand-binding domain, the pseudokinase domain, and the adenylate and guanylate cyclase catalytic domain). Thus, it isn't clear whether they may differ in their function. Our search of the literature and also NCBI's "Protein Domains and Macromolecular Structures" search tool did not uncover any relevant information in this regard about guanylate cyclases in general nor about CG10738 in particular.

For the UAS-EHR construct, the cDNA we amplified was identical to isoform called "C" in Flybase. We have added this detail to the Materials and Methods (lines 613-614).

Second, regarding the diversity and functional heterogeneity of different EHR-expressing cell types (Tables 1 and 2): I was interested to see that nsyb Gal4 KD of EHR produced strong effects in pupal but not larval ecdysis. A very similar pattern was found with btl-Gal4. But remarkably, btl-Gal4 could drive strong rescue of EHR mutants in larval stages stages, but not in pupae. Do the authors have any speculation as to why certain EHR bearing cells (btl-expressing) might be sufficient, while not required, for EHR function at some stages? Compensatory functions for btl and nsyb populations? Was the rescue also tried with n-sybGal4?

In general, we observed that pupal ecdysis was much more sensitive to genetic manipulations (more sensitive to RNAi and more difficult to rescue) than were the larval ecdyses. This is probably because to pupate the animal has to evert its head and appendages in addition to shed its old cuticle, which may require additional cells and/or higher expression of EHR in key EH targets. Thus, if only a little EHR expression were required for larval ecdyses, then knockdown using a weak driver may produce few, if any, deficits, and rescue would be expected to be strong because the small amount of EHR driven by the weak driver would be sufficient to confer normal function. At pupation, by contrast, the same weak driver would produce strong deficits because even partial knockdown would reduce EHR expression to levels below the threshold for correct cellular function. Likewise, rescue at the pupal stage would be poor because the low levels of UAS-EHR expression would now be insufficient to restore function.

We would also note that "necessary” and “sufficient” are not different sides of the same experimental coin because “necessary” depends on the effectiveness of RNAi knockdown, whereas “rescue”/sufficiency is based on the effectiveness of targeted expression of EHR in rescuing the mutant phenotype. We attempted to carry out directed CRISPR/Cas9 gene knockouts in order to properly address the question of “necessity”, but the knockouts were not sufficiently effective to be useful.

We did not include nsyb-GAL4 as a driver for the rescue experiments, but other neuronal drivers (e.g., 386Y), provide an exemplar of a neuronal driver for which knockdown caused failure only at pupation and no rescue at this stage.

Reviewer #2:

Major comments:

In response to my previous (major) comment regarding Figure 7 (now Figure 8), the authors explained that treating the brain with both ETH and EH during the in vitro imaging experiments is technically infeasible. However, I believe the authors can evaluate the synergistic effects of ETH and EH on CCAP neurons by investigating Ca^{2+} activity in CCAP cells expressing EH-R-RNAi or ETH-R-RNAi during ETH-induced fictive ecdysis behavior.

We appreciate this suggestion, but we expect that the interpretation of such an experiment would be complicated due to the contribution of indirect actions of ETH and EH on the CCAP neurons. For instance, in Mena et al., (2016; https://doi.org/10.7554/eLife.19686) we showed that inhibitory GABA-ergic neurons are likely activated by ETH, which then prevent CCAP neurons from firing until their response subsides. Such indirect actions would make it very difficult to tease out the contributions of EH and ETH to the response of CCAP neurons. Thus, we believe that investigating the synergistic actions of EH and ETH will be best taken up later as part of a broader investigation.

Minor comments:

1. The knockdown efficiency of EH-R-RNAi was not evaluated. I believe it is necessary to evaluate the EH-R knockdown efficiency using EH-R>EH-R-RNAi in the brain or CNS.

Thank you for the suggestion. However, this experiment cannot be done because the EHR-GAL4 driver is an insertion of GAL4 in the EHR gene, so its activity depends on the expression of EHR. Thus, driving an EHR-RNAi using this driver will affect GAL4 expression, rendering the results uninterpretable. The closest we could come to this experiment would be to use the tubulin-GAL4 driver, as it is likely expressed in all EHR cells. As shown in Tables 1 and 2, knockdown of EHR using this driver caused defects as severe as those obtained using the EHR mutants. Thus, knockdown using this EHR-RNAi is clearly effective. Furthermore, we consider the phenotypic readout of knockdown to be a more relevant measure of knockdown efficiency than is measuring the change in gene expression. Indeed, the relationship between the level of gene expression and phenotype differs for different genes, with a knockdown of even 80% causing the expression of a mutant phenotype for some genes but not for others. Thus, in this case, phenocopying a severe mutant phenotype is much more informative than measuring the efficiency of EHR knockdown in terms of changes in levels of gene expression.

2. Figure 1: "F" and "G" should be labeled "E" and "F," respectively.
Many thanks for pointing out this error; it has now been corrected.

3. Supplemental Fig. 1 shows mimic cassette insertion, which was not used or mentioned in the manuscript.
Many thanks for pointing this out; this insertion has been removed from the map.

4. Line 220: "C, F" should be "C-F."
Many thanks for pointing out this error; it has now been corrected. (line 222)

Reviewer #3:
The authors have responded to all of my questions and suggestions very well.
This includes providing information about production and quantification of eclosion hormone and clarification of various other issues.
We were happy to read this!

A couple of issues remain:

1. One was intrigued by the authors' response to Reviewer 2 regarding the narrow window of Inka cell responsiveness to EH. The authors state that "Inka cells did not respond to EH unless they were challenged less than 10 minutes before ecdysis (results shown in Fig. 1)". In fact, exposure to EH in this figure occurs only at a single time point: dVP (double vertical plates). According to Park et al., (2002); Ref 32, dVP occurs 10 min prior to tracheal collapse (TC), 15 min before pre-ecdysis, and ~25 min prior to ecdysis. Are the authors stating that Inka cells are insensitive to EH prior to dVP? Although Fig. 1 does not present any data in support of this claim, it is a very important point. Would it be possible to add some negative data on Inka cell responsiveness prior to dVP in Fig. 1 to solidify this finding?
In our hands Inka cells were not responsive to *in vitro* EH stimulation until very close to the normal time of ecdysis. For a long time we thought that the EH we had made was just inactive (its synthesis was not devoid of problems, as described in Materials and Methods). Fortunately, we eventually tested trachea from animals closer to ecdysis and it then worked. But the reviewer is correct that Figure 1 indicates that the animals used for *in* vitro stimulation were at the dVP stage. We have now revised the labeling of Figure 1, added a note to the figure legend regarding the stage of the animals used for *in vitro* stimulation (lines 153-155) as well as to the Materials and Methods section (lines 639-643).

2. Regarding the figure legend of the revised Supplementary Video 1, reference to Fig 7 should now be to Fig 8, since two figures have been exchanged in the revised manuscript.
Many thanks for pointing out this error; it has now been corrected.
